# Performance in information processing speed is associated with parietal white matter tract integrity in multiple sclerosis

**DOI:** 10.3389/fneur.2022.982964

**Published:** 2022-11-04

**Authors:** Matthias Grothe, Katharina Jochem, Sebastian Strauss, Sönke Langner, Michael Kirsch, Kai Hoffeld, Iris Katharina Penner, Guy Nagels, Kai Klepzig, Martin Domin, Martin Lotze

**Affiliations:** ^1^Department of Neurology, University Medicine Greifswald, Greifswald, Germany; ^2^Institute for Diagnostic Radiology and Neuroradiology, University Medicine of Greifswald, Greifswald, Germany; ^3^Department of Neurology, Medical Faculty, Heinrich Heine University Düsseldorf, Düsseldorf, Germany; ^4^COGITO Center for Applied Neurocognition and Neuropsychological Research Düsseldorf, Düsseldorf, Germany; ^5^Department of Neurology, Inselspital, Bern University Hospital, University of Bern, Bern, Switzerland; ^6^Center for Neurosciences, Vrije Universiteit Brussel, Brussels, Belgium; ^7^National MS Center Melsbroek, Steenokkerzeel, Belgium; ^8^Functional Imaging, Institute for Diagnostic Radiology and Neuroradiology, University Medicine of Greifswald, Greifswald, Germany

**Keywords:** multiple sclerosis, cognition, SDMT, diffusion tensor imaging, brain mapping

## Abstract

**Background:**

The Symbol Digit Modalities Test (SDMT) is most frequently used to test processing speed in patients with multiple sclerosis (MS). Functional imaging studies emphasize the importance of frontal and parietal areas for task performance, but the influence of frontoparietal tracts has not been thoroughly studied. We were interested in tract-specific characteristics and their association with processing speed in MS patients.

**Methods:**

Diffusion tensor imaging was obtained in 100 MS patients and 24 healthy matched controls to compare seed-based tract characteristics descending from the superior parietal lobule [Brodman area 7A (BA7A)], atlas-based tract characteristics from the superior longitudinal fasciculus (SLF), and control tract characteristics from the corticospinal tract (CST) and their respective association with ability on the SDMT.

**Results:**

Patients had decreased performance on the SDMT and decreased white matter volume (each *p* < 0.05). The mean fractional anisotropy (FA) for the BA7A tract and CST (*p* < 0.05), but not the SLF, differed between MS patients and controls. Furthermore, only the FA of the SLF was positively associated with SDMT performance even after exclusion of the lesions within the tract (*r* = 0.25, *p* < 0.05). However, only disease disability and total white matter volume were associated with information processing speed in a linear regression model.

**Conclusions:**

Processing speed in MS is associated with the structural integrity of frontoparietal white matter tracts.

## Introduction

Cognitive impairment is common in up to 40%−50% of patients with multiple sclerosis (MS) (1) and has been associated with both gray (2, 3) and white matter (4, 5) pathologies. Previous studies related to white matter abnormalities associated with cognitive impairment in MS have focused on lesion load (4), lesion location (5), or whole-brain white matter tract integrity (6). With these approaches, several fiber tracts, such as the fornix, corpus callosum, thalamic radiation, and superior longitudinal fasciculus (SLF), have been related to cognitive disturbances (7, 8).

The Symbol Digit Modalities Test (SDMT) assesses information processing speed and working memory, discriminating patients from healthy controls with high sensitivity (1, 9, 10). When applying functional magnetic resonance imaging (fMRI) to investigate the underlying neural resources, a network consisting of frontal (Brodman area [BA] 6 and 9), parietal (BA7), occipital (BA17), and medial posterior cerebellar (declive) regions have been identified (11–13). Based on the involvement of a widespread functional network in processing speed, the SLF, and especially its subdivisions SLF1 and SLF2, is presumably important because this tract bundle connects the super parietal lobule with the superior and middle frontal areas (14–16).

A recent fMRI approach of the oral version of the SDMT also emphasized the role of the superior parietal lobe (SPL), especially BA7A, for SDMT performance (17). This area is particularly involved in spatial attention and visual working memory (18, 19), which represent key components of the SDMT (20). Anatomically, BA7A is structurally interconnected with frontal, temporal, and brainstem areas, at least in part *via* the SLF (15), again highlighting the importance of this white matter tract bundle for cognition in MS.

Here, we investigated structural white matter alterations in MS patients to better understand the role of specific parietal white matter tracts, especially the SLF, and their associations with ability on the SDMT. We focused on tract integrity as quantified by fractional anisotropy (FA) using diffusion tractography (DTI) on diffusion weighted imaging (DWI), as FA is a highly sensitive, early, diffusion tensor-derived metric for demyelination (21). In the literature, different DTI methods are used – either in a whole brain approach, called tract based spatial statistics (6), or with a more hypothesis driven, regional approach. Based on the literature that highlights the importance of the SLF and BA7A, we chose a regional-based approach to associate the clinical impairment in the SDMT with white matter tract pathology, using a predefined probabilistic region-of-interest of the SLF and by performing probabilistic tractography originating in BA7A. As a reference tract we selected the corticospinal tract (CST), the integrity of which has been associated with motor, but not cognitive, performance (22). Whole-brain and tract-specific metrics were compared between MS patients and healthy controls.

We further analyzed the tract metrics for the whole tract and after exclusion of the lesions within the tract because we were especially interested in an association with the so-called normal appearing white matter (NAWM) tract alterations, which are also accompanied by a reduction in FA in MS (23, 24). In a final step, we performed correlation and linear regression analyses to investigate clinical and imaging variables and their association with SDMT.

## Methods

### Participants

A total of 100 MS patients were enrolled in this study [70 females, mean age 44.3 years, median Expanded Disability Status Scale (EDSS) 2.0]. All MS patients fulfilled the criteria for multiple sclerosis according to the 2017 McDonald criteria (25). Exclusion criteria were an acute relapse or steroid treatment within the previous 3 months and another central neurological disease. Twenty-four healthy controls (HCs) were added as a control group without any neurological or psychiatric disorder. The study was approved by the Ethics Committee of the Medical Faculty of the University of Greifswald (BB028/13) and all participants provided informed consent. Demographics are summarized in Table 1.

**Table 1:** Group characteristics.

	**Patient group**	**Control group**	**Test statistics**
*N*	100	24	
Age (years)	44.1 ± 12.5	41.1 ± 11.56	*t* = 1.1; *p* = 0.28
Sex (male/female)	30/70	10/14	χ^2^ = 2.8; *p* = 0.12
Education (years)	14.5 ± 1.8	14.2 ± 2.8	*t* = 0.59; *p* = 0.55
Disease duration (years)	8.9 ± 7.0		
Disease course (RRMS/SPMS)	93/7		
EDSS	2.0 (0–7)		
zSDMT	−0.47 ± 1.3	0.12 ± 1.2	*t* = 2.04; *p* = 0.04
Gray matter volume (cm3)	615.1 ± 72.8	644.4 ± 72.1	*t* = −1.77; *p* = 0.08
White matter volume (cm3)	498.5 ± 65.8	546.3 ± 63.2	*t* = −3.22; *p* = 0.002
Lesion volume (cm3)	8.5 ± 8.0	2.0 ± 1.2	*t* = 7.8; *p* <0.001
FA, SLF	0.436 ± 0.02	0.439 ± 0.02	*t* = 0.4; *p* = 0.69
FA, BA7A tract	0.429 ± 0.04	0.447 ± 0.03	*t* = 2.2; *p* = 0.03
FA, CST	0.455 ± 0.02	0.465 ± 0.02	*t* = 2.3; *p* = 0.02
Lesion overlap (%): SLF	1.019 ± 1.344	n/a	
Lesion overlap (%): BA7A tract	1.951 ± 1.509	n/a	
Lesion overlap (%): CST	1.683 ± 1.281	n/a	

Values are given as mean ± standard deviation or median (range) unless otherwise noted.

### Neurological and neuropsychological examination

Each patient was investigated clinically and neuropsychologically with respect to clinical disability (EDSS) (26), depression [Beck Depression Inventory-II (BDI-II)] (27), fatigue [Fatigue Scale for Motor and Cognitive Functions (FSMC)] (28), and information processing speed (SDMT).

**Figure 1:**
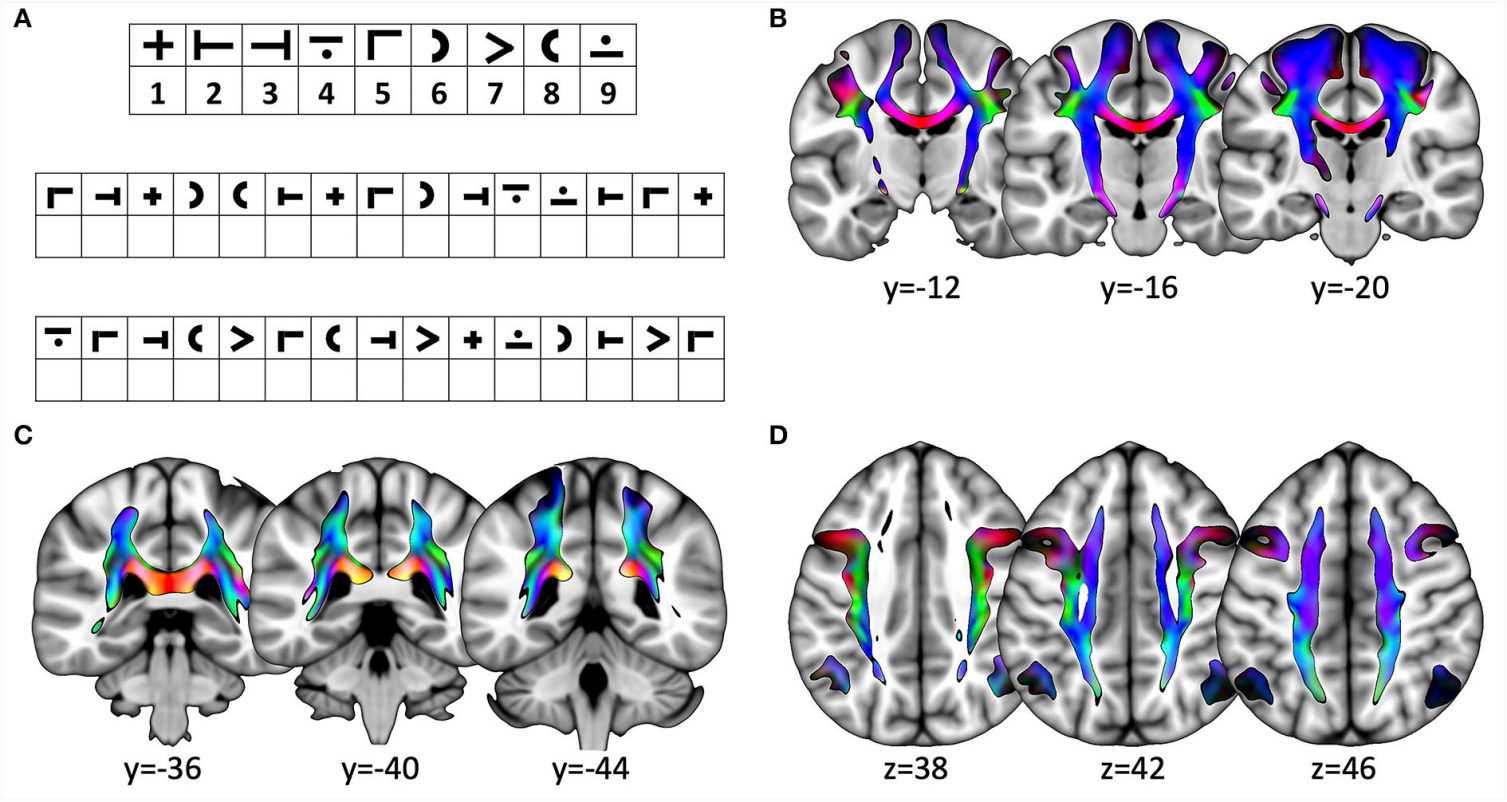
**(A)** Example for the Symbol Digit Modalities Test (SDMT). **(B–D)** Slices showing the three tracts from the diffusion-weighted imaging data for all participants. The direction of tractography is encoded in standardized colors: *z*, blue; *y*, green; *x*, red. **(B)** coronal slices depicting the tract originating in M1; **(C)** coronal slices showing the tract originating in BA7A; and **(D)** axial slices showing SLF. Slice position is indicated in the respective direction below slice.

The control group was investigated with only the SDMT. Figure 1A demonstrates an example of the SDMT. All clinical assessments and MRI measurements were performed within 2 weeks.

### MRI data acquisition

MRI was performed on a 3-T scanner (Magnetom Verio, SIEMENS, Erlangen) using a 32-channel head coil. The standard imaging protocol in all patients included a sagittal T1-weighted 3D-Magnetization Prepared Rapid Acquisition with Gradient Echoes (MPRAGE) sequence (TR: 1,690 ms; TE: 2.52 ms; TI: 900 ms; flip angle: 9°; matrix: 256 × 256; 176 slices; voxel size 0.98 × 0.98 × 1 mm), a 3D-T2-FLAIR sequence [TR: 5,000 ms; TE: 388 ms; TI: 1,800 ms; matrix: 512 × 512 (k-space interpolation); 160 slices; voxel size 0.49 × 0.49 × 1 mm], and a Siemens- Multi- Directional Diffusion Weighted (MDDW) sequence [TR: 10,900 ms; TE: 107 ms; flip angle: 90°; matrix: 128 × 128; voxel size: 1.8 × 1.8 × 2 mm; 70 slices; 1 × unweighted volume (*b* = 0); 64 × diffusion-weighted volumes (*b* = 1,000)].

### MS lesion segmentation

Lesions were segmented by the lesion prediction algorithm (LPA) as implemented in LST toolbox version 3.0.0 (www.statistical-modeling.de/lst.html) for statistical parametric mapping (SPM; Wellcome Center, London, UK) (29). The LPA classifier was trained using a logistic regression model as described in detail elsewhere, providing an estimate for the lesion probability of each voxel (29). The 3D-T2- fluid-attenuated inversion recovery (FLAIR) sequence is sufficient as an exclusive source for lesion segmentation when using this prediction algorithm. The resulting lesion maps were visually inspected for gross deviations by an expert (MG), and no further correction was needed. The final maps were subsequently used as exclusion masks for later extraction of FA and to calculate a possible overlap for a quantification of lesion load of certain white matter tracts.

### Image processing

The diffusion-weighted data were corrected for eddy current and motion-related artifacts [FSL eddy_correct (v6.0.1)], followed by appropriate correction of the diffusion gradient vector table. Afterwards, the diffusion tensor was calculated by least-square fitting (FSL dtifit) and the usual DWI metrics, such as FA. A spatial transformation was calculated from the diffusion image space into the MNI template space by generating a group template (antsMultivariateTemplateConstruction2, Advanced Normalization Tools v3.0.0.0.dev21-g1d890) based on the FA images of all patients and healthy subjects. This group template was then registered to the MNI 152 ICBM 6th gen. template brain using ANTs SyN (30).

The inverse of the merged registration (MNI template : group template : single subject) was used to transform regions-of-interest (ROIs) of the Juelich histological atlas (SPL, BA7A, left and right hemisphere) (31), the Brainnetome Atlas (primary motor cortex, M1, left and right hemisphere) (32), and the human XTRACT atlas (SLF parts 1 and 2, left and right hemisphere) (33) from the MNI template space into individual subject space.

Next, separately for each ROI and hemisphere, unconstrained structural connectivity was generated using probabilistic tractography FSL's probtrackx (34). For that purpose, FSL's bedpost (35) was applied to calculate the fiber orientation density function (FODf) from the diffusion MRI for each voxel. The FODf can then be randomly sampled to extract principal diffusion directions in each voxel. Starting at a seed voxel of a ROI these directions can be followed and put together to a streamline. As the FODf can contain multiple principal diffusion directions, a seed voxel will “spawn” many thousand different streamlines depending on the selected direction in each voxel. This process results in a frequency map in which each voxel encodes the number of valid streamlines running through that voxel. In addition, as the XTRACT atlas already contains these frequency maps, therefore tractography was not needed and the extracted ROIs were used as a generic tractogram.

Finally, the intensity values of each resulting tractogram were numerically normalized to 1 by dividing each voxel value by the highest voxel value of the respective tractogram and then used to calculate a weighted mean FA value for each tractogram in a way that each voxel's FA value was scaled (weighted) by the corresponding tractogram's normalized frequency.

For visualization purposes (see Figures 1B–D), each calculated tractogram was transformed into the MNI space and all tractograms belonging to the same ROI were averaged. This procedure was also applied to the individual lesion maps, resulting in an average lesion map in the MNI space.

In order to quantify the gray and white matter volumes, the CAT12 Toolbox (Christian Gaser, https://neuro-jena.github.io/cat/) for SPM (SPM12; Wellcome Department of Cognitive Neurosciences, London, UK) was used. As the CAT12 Toolbox is capable of identifying white matter hyperintensities, the lesions were removed from the calculation of white matter volume.

### Statistical analysis processing

All statistical testing was performed using SPSS version 25. Descriptive statistics were performed according to the data using means with standard deviations or medians with ranges. Basic assumptions of normal distribution were assessed as recommended both visually and by the Shapiro-Wilk test. The raw score for the SDMT was corrected for age and education level based on the German validation study, resulting in SDMT z-scores (zSDMT) (36). Group differences between patients and HCs were assessed using the Student's *t*-test or Mann–Whitney *U*-test. Differences between each tract (SLF, BA7A tract, CST) with or without lesion masking were determined using paired *t*-tests.

To investigate the associations between zSDMT and imaging data, Pearson or Spearman correlations were computed depending on their normal distribution. A stepwise multiple linear regression model was finally calculated with zSDMT as the dependent variable and clinical (disease duration, EDSS, FSMC, BDI) and imaging (gray matter volume, white matter volume, lesion volume, FA SLF, FA BA7A-tract, FA CST) variables as independent variables.

A significance level of 0.05 was used and *p*-values adjusted by Benjamini–Hochberg's procedure in order to correct for multiple comparisons.

## Results

### Clinical characteristics

In the MS patient group, the mean disease duration was 7.1 years, median EDSS 2.0, mean zSDMT −0.47, mean FSMC 54.8, and mean BDI 9.7. In this group, 11.8% of patients were not treated, 62.3% were treated with first-line disease-modifying drugs (DMDs), and 25.7% were treated with second-line DMDs. The patient group performed worse than the control group on the SDMT (*t* = 2.04; *p* = 0.04). Group comparisons for MS patients and healthy controls are summarized in Table 1.

### Imaging characteristics

**Figure 2:**
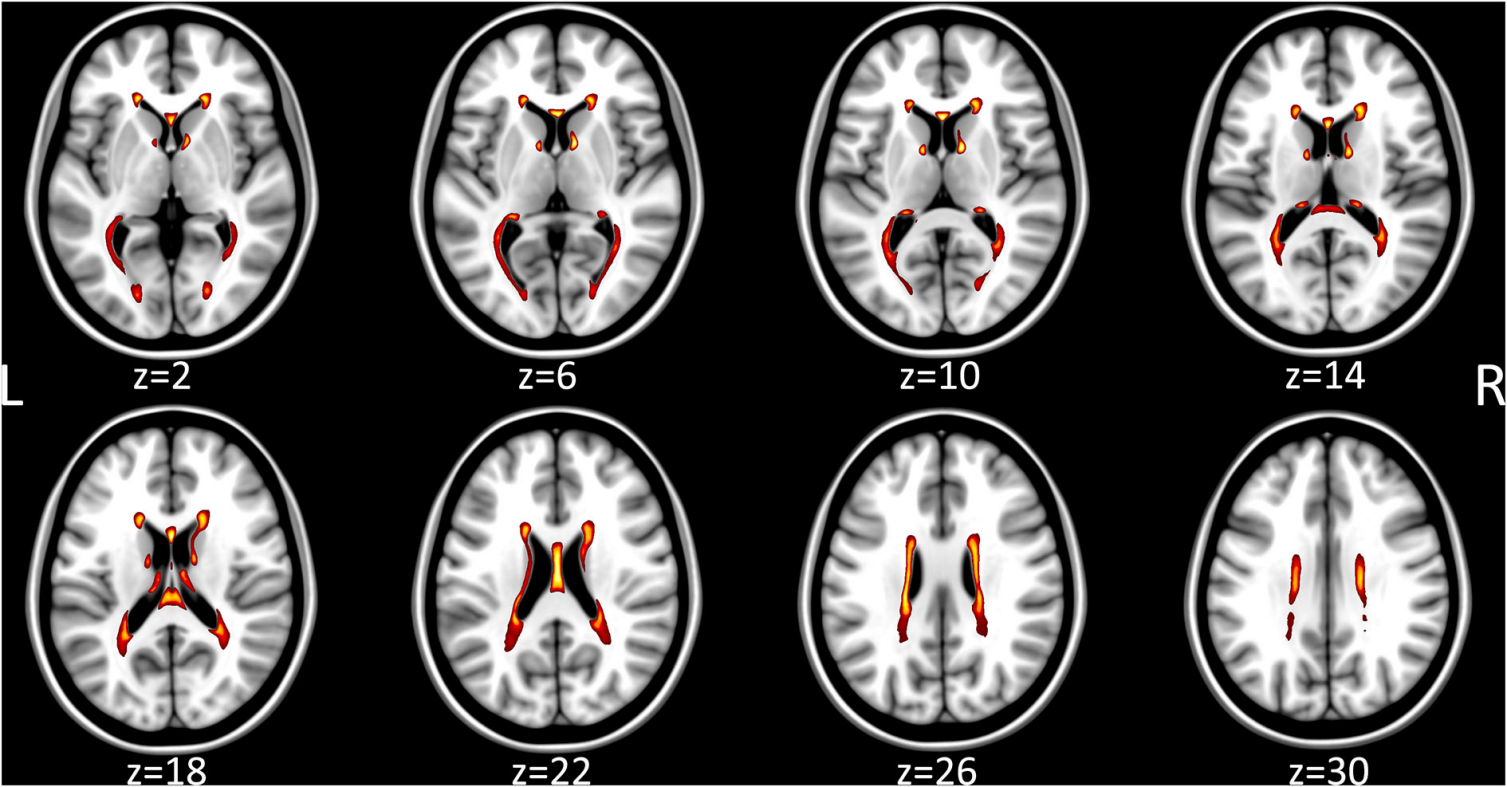
Heatmap of the individual multiple sclerosis lesion maps, which were transformed into MNI template space, averaged voxel-wise, thresholded to 25% and color-coded (white 100% overlap, red 25% overlap). Axial slice position is indicated below the MRI respectively.

Structural data revealed reduced white matter volume and higher lesion volume for MS patients compared to HCs (see Table 1, Figure 2). Comparison of the tracts without lesion exclusion revealed that FA for the SLF did not differ between patients and controls (*t* = 0.4, *p*_FDR_ = 0.7), whereas BA7A tract and CST showed lower FA in patients than in controls (BA7A tract: *t* = 2.2, *p*_FDR_ = 0.045; CST: *t* = 2.3, *p*_FDR_ = 0.036). Lesion exclusion did not have an impact on the main findings (SLF: *t* = 0.4, *p*_FDR_ = 0.7; BA7A *t* = 2.8, *p*_FDR_ = 0.024, CST: *t* = 2.4, *p*_FDR_ = 0.036).

For MS patients, FA of the SLF and BA7A tract, but not for the CST, differed significantly between the analysis with and without lesion exclusion (SLF: *t* = 2.9, *p*_FDR_ = 0.0225; BA7A tract: *t* = 4.2, *p*_FDR_ = 0.009; CST: *t* = 0.8, *p*_FDR_ = 0.5).

### Correlation between clinical and imaging data

**Figure 3:**
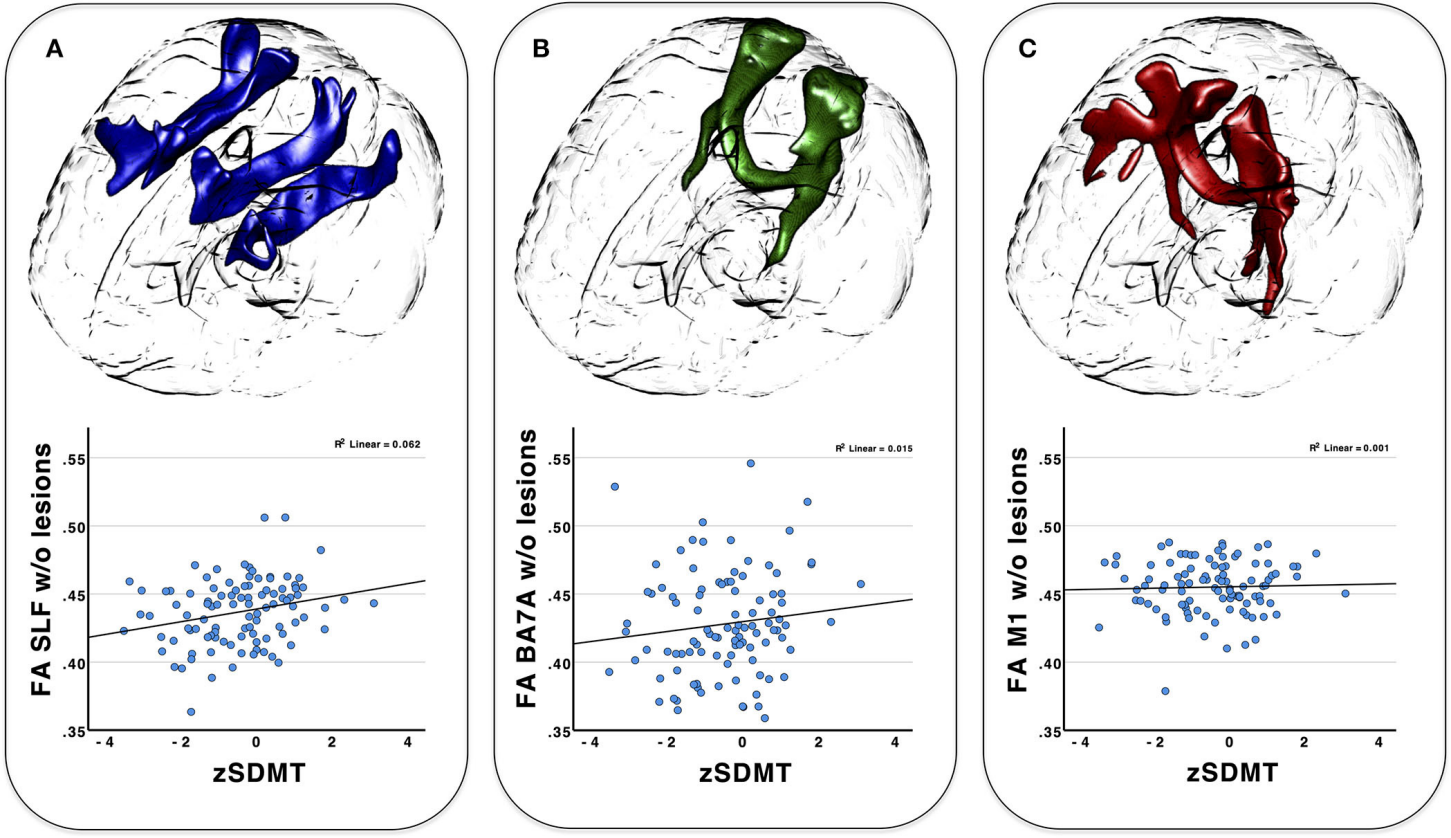
3D-tractogram of all three tracts investigated (top) and the plotted correlation of behavioral data (SDMT) with the weighted mean FA of the tracts after lesion exclusion (bottom). **(A)** superior longitudinal fasciculus (SLF); **(B)** BA7A tract; **(C)** corticospinal tract (CST).

Visual inspection and the Shapiro-Wilk test revealed a normal distribution for zSDMT and FA for each tract. For MS patients, Pearson correlations between zSDMT and FA revealed a significant association of the SLF (*r* = 0.246, *p*_FDR_ = 0.042), but not the BA7A tract (*r* = 0.113, *p*_FDR_ = 0.4) or the CST (*r* = 0.033, *p*_FDR_ = 0.75). Plots and tract visualization are provided in Figure 3. The association of FA SLF and zSDMT remained significant after lesion exclusion (*r* = 0.25, *p*_FDR_ = 0.04). zSDMT and FA of tracts from the HCs (each *p* > 0.2) showed no relevant association.

### Linear regression analysis

The stepwise linear regression model with zSDMT as a dependent variable revealed EDSS (*β* = −0.365, *p* < 0.001) and white matter volume (*β* = 0.223, *p* = 0.02) as significant independent variables (*R*^2^ = 0.221, *p* < 0.001) for the MS patients.

## Discussion

With our hypothesis-driven approach, we demonstrated a positive association between processing speed performance and white matter tract integrity for the SLF, which emphasizes the importance of intact frontoparietal structural connectivity for information processing speed performance. The significance remaining after lesion exclusion also indicates that the tract integrity depends not only on white matter lesions, but also on the NAWM.

For MS, several studies have investigated the relationship between white matter integrity and cognition, especially for processing speed (37–39). Cognitively impaired MS patients have been shown to have decreased FA values compared to unimpaired patients and controls at both the whole-brain level (37) and within several anatomically defined white matter regions, especially the corpus callosum, SLF, and internal capsule (7, 8).

Here, we focused on white matter tracts based on existing imaging studies on information processing speed performance in MS patients (11, 17). Based on the literature, BA7A is a crucial area for spatial attention and visuomotor control (15, 19) and of high importance for performance on the SDMT (17). Using probabilistic tractography in our cohort of 100 MS patients, we demonstrated that the integrity of this tract differs between MS patients and HCs but in contrast to our assumptions, no significant association was demonstrated between tract integrity and SDMT performance in the MS patients. We defined the tract bundle based on the anatomical maps of BA7A, resulting in a structural network merging with the posterior corona radiate, splenium and body of the corpus callosum, SLF, and the posterior and retrolenticular part of the internal capsule. This widespread structural network connecting frontal, temporal, and cerebellar regions (15) may be only partially involved in processing speed, resulting in low specificity of this predefined tract for the SDMT.

The tract originating in BA7A largely merges into the SLF. The SLF, and especially its subdivisions SLF I and SLF II, are mainly interconnecting frontal and parietal regions (16). The mean FA value of the SLF in our cohort did not differ between the groups, but the association between the mean FA and the individual SDMT score revealed a significant, albeit not strong correlation. Interestingly, this significance remained even after exclusion of the lesions within the tract. A few studies have suggested a role of the SLF in cognition in MS (8, 40), but these approaches did not test for specific tracts and did not control for lesions within the tract. Our data in this way confirm the importance of parietal white matter tract bundles for cognition in MS, and highlight the contribution of the NAWM tract integrity on clinical impairment (41, 42). We are aware that several other studies did not find any associations between SDMT and frontoparietal tracts (43, 44), but especially as we used a hypothesis driven approach, we highlight the importance of these tract bundles for cognition in MS.

In MS patients, myelin content and axon count within the NAWM correlate with FA (45, 46). Our data suggest that these alterations may lead to clinical impairment even if FA of the tract does not differ between the patients and controls, and that the alterations within the SLF are important for the clinical impairments.

Using a linear regression model, we demonstrated that disability and total white matter volume, not the integrity of presumed tracts, are the most important variables for processing speed. These findings were unexpected, especially as it is somewhat different from other studies (47). The disability and white matter volume as significant predictors for processing speed in our mildly disabled cohort highlight the importance of an intact structural network that extends beyond the tracts investigated in our study. Therefore, the contribution of parietal white matter tracts like the SLF should be considered in a larger structural network. Another possible explanation is that the structural alteration of the parietal tact was not so severe, especially for the SLF, as the FA was not significant different between the patients and the controls. The relationship with disease disability conceptualized with the EDSS, though known in principle (1, 38), was also somewhat surprising because our cohort was generally only mildly impaired with a median EDSS of 2.0. Both clinical and structural variables are contributing differentially on cognitive impairments in MS depending on increasing disability (48, 49), and future research should also focus especially on the NAWM pathology and their role in cognitive impairment in MS. In addition, as we focused on white matter alterations and their importance in processing speed, we only added the total gray matter volume as an independent variable, but we are aware of the importance of gray matter, especially deep gray matter volume, on cognition (8, 38). Other clinical variables in our cohort of moderately fatigued and minimally depressed patients could not explain the additional variance in our model.

Our study has several limitations. The main limitation is the selection of only a limited number of tracts. Keeping in mind that cognitive speed is dependent on a network of interacting neural resources and not limited to one or two structures, our hypothesis-driven approach showed the expected contribution of the SLF, but not the BA7A tract, in task ability. In addition, the level of disability in our cohort was rather low, which might affect the generalizability of our results. Furthermore, the used lesion segmentation algorithm only detects white matter hyperintensities, that cannot definitely be declared as MS related or of other origin like vascular. Future research has to combine functional and structural connectivity measurements to confirm our results in independent samples. Finally, the acquired diffusion data lacks technical merits, as at the time of acquisition only older protocols and sequences were available with a rather long echo time, only one b0-image and no inverse phase-encoded b0-image (or whole dataset) for distortion or noise correction.

In conclusion, we demonstrated that the structural integrity of the NAWM parts of the SLF is associated with processing speed in mildly impaired MS patients. The structural alterations also in NAWM should be kept in mind for future research into the underlying processes of information processing speed in MS as well as for therapeutic approaches such as noninvasive brain stimulation.

## Data availability statement

The raw data supporting the conclusions of this article will be made available by the authors, without undue reservation.

## Ethics statement

The studies involving human participants were reviewed and approved by Ethics Committee of the Medical Faculty of the University of Greifswald. The patients/participants provided their written informed consent to participate in this study.

## Author contributions

MG and ML contributed to the study conception and design. MG and MD performed material preparation, data collection, and analysis. MG written the first draft of the manuscript. KK and KH measured and tested healthy controls. SL and MK helped with MRI of MS patients. All authors commented on previous versions of the manuscript, read, and approved the final manuscript.

## Conflict of interest

MG received honoraria or speaking fees from Biogen, Celgene, Merck Serono, Novartis, Roche, Sanofi Genzyme, and Teva. IP has received honoraria for speaking at scientific meetings, serving on scientific advisory boards, and consulting activities from Adamas Pharma, Almirall, Bayer Pharma, Biogen, BMS, Celgene, Desitin, Sanofi-Genzyme, Janssen, Merck, Novartis, Roche, and Teva. She has also received research support from the German MS Society, Celgene, Novartis, Roche, and Teva. ML is a paid editor for the Thieme Verlag. The remaining authors declare that the research was conducted in the absence of any commercial or financial relationships that could be construed as a potential conflict of interest.

## Publisher's note

All claims expressed in this article are solely those of the authors and do not necessarily represent those of their affiliated organizations, or those of the publisher, the editors and the reviewers. Any product that may be evaluated in this article, or claim that may be made by its manufacturer, is not guaranteed or endorsed by the publisher.
